# Addressing Academic Stress in Secondary Education: A Systematic Review of Intervention Approaches and Related Outcomes

**DOI:** 10.3390/healthcare14193346

**Published:** 2026-10-08

**Authors:** Miriam Catalina González-Afonso, Raquel García-García, Iris Alexia Hernández-González, David Pérez-Jorge

**Affiliations:** 1 Department of Didactics and Educational Research, Faculty of Education, University of La Laguna, 38204 San Cristóbal de La Laguna, Spain; mcglez@ull.edu.es (M.C.G.-A.); ihernang@ull.edu.es (I.A.H.-G.); 2 School of Doctoral and Postgraduate Studies, University of La Laguna, 38200 San Cristóbal de La Laguna, Spain; raquel.garcia.12@ull.edu.es

**Keywords:** academic stress, secondary school students, academic demands, school-based interventions, adolescent mental health, psychological well-being

## Abstract

**Background/Objectives:** Academic stress is associated with a range of adverse psychological and educational outcomes among secondary school students, yet evidence on interventions addressing academic stress and related academic demands remains heterogeneous. This systematic review aimed to synthesise this evidence and examine the intervention approaches, delivery modalities, and outcomes reported. **Methods:** The review followed PRISMA 2020 guidelines and was registered in PROSPERO (CRD420261476775). Web of Science, Scopus, PsycINFO, and PubMed were searched for studies published between 2015 and 2026, with the final search conducted on 12 August 2026. During full-text eligibility assessment, the registered outcome criterion was expanded to include related psychological or educational outcomes in studies addressing academic demands; this post-registration deviation is reported explicitly in the review. Two reviewers independently screened studies, and methodological appraisal was conducted using JBI and MMAT tools. Given the heterogeneity of study designs, interventions, outcomes, and effect-size metrics, findings were synthesised narratively. **Results:** Eighteen studies were included. Five intervention approaches were identified: socio-emotional and life-skills programmes (*n* = 4), cognitive-behavioural and rational-emotive interventions (*n* = 5), mindfulness- and acceptance-based interventions (*n* = 4), stress-management and mindset interventions (*n* = 3), and organisational approaches (*n* = 2). Academic or school-related stress was assessed directly as an intervention outcome in four studies, while the remaining 14 examined related psychological and educational outcomes and constituted a secondary, non-comprehensive extension of the evidence base. Favourable findings were reported across several intervention approaches, but effects varied according to the outcome assessed, and direct evidence for academic or school-related stress was less consistent than evidence for related psychological and educational outcomes. Most interventions were delivered face-to-face, while evidence on digital, blended, and organisational approaches was limited. **Conclusions:** The available evidence suggests that several intervention approaches may be useful for addressing academic stress or related responses to academic demands among secondary school students. However, direct evidence for academic or school-related stress remains limited and heterogeneous, and differences in study designs, measures, and effect-size metrics preclude conclusions about the comparative superiority of any one approach.

## 1. Introduction

Adolescence is a developmental period characterised by substantial physical, cognitive, emotional, and social changes that coincide with increasing educational demands [1,2]. During this stage, students must manage academic expectations alongside personal, family, and social challenges, while mental health problems represent an important concern across adolescent populations [3]. Within this context, school experiences and academic demands constitute relevant sources of psychological strain and may affect both students’ well-being and their educational functioning [4,5,6]. Academic stress is the central construct examined in this review and is distinguished from related psychological and educational outcomes that may occur in response to academic demands but do not constitute equivalent measures of academic stress.

Within the school context, students assume a role involving a range of responsibilities and demands and are required to cope with different situations, including academic expectations [5] and personal, family, social, and contextual factors that may generate tension [1], including those arising from the COVID-19 pandemic [6]. Academic stress can be understood as a systemic process that emerges when students perceive academic demands—such as workload, schedules, examinations, or performance expectations—as exceeding their available coping resources [5]. This imbalance may be accompanied by psychological or physical symptoms and may prompt students to mobilise coping strategies in an attempt to restore equilibrium. Academic stress therefore reflects not simply the presence of educational demands, but students’ responses to those demands and their perceived capacity to manage them.

Among secondary school students, school-related stress has been reported across different cultural contexts and demographic groups [7,8,9], with assessments and other performance-related demands representing particularly relevant sources of stress [10,11,12,13,14]. Although moderate stress may sometimes accompany adaptive engagement with academic demands, persistent or excessive stress has been associated with poorer psychological and educational outcomes [7,10,15,16,17,18,19]. Reported correlates include depressive and anxiety symptoms [13,16,19,20,21,22,23], psychological distress [15,20], school burnout [21], reduced motivation and confidence [13,19], and lower life satisfaction [18,24]. These findings underline the relevance of academic stress not only as an educational issue but also as a concern for adolescent mental health and well-being.

Although academic stress is the central construct examined in this review, it is associated with a broader range of psychological and educational outcomes, including test anxiety [14], psychological distress and academic self-efficacy [17], depression and school burnout [21], and academic engagement [25]. These constructs may arise in relation to academic demands or change following interventions that address those demands, but they should not be regarded as interchangeable measures of academic stress. Accordingly, the present review distinguishes throughout between outcomes that directly assess academic or school-related stress and related psychological or educational outcomes.

Given these consequences, schools provide an important setting for preventive and supportive interventions aimed at helping adolescents manage academic demands and promoting psychological well-being [26,27,28]. Such interventions may operate at different levels, including the development of individual coping and emotional-regulation skills, structured psychological or psychoeducational programmes, school guidance and support, and organisational changes intended to modify sources of academic burden. Organisational and policy-level approaches were therefore included because they may address the academic demands that contribute to stress rather than students’ individual responses to those demands. They were considered as a distinct intervention level and were not assumed to be directly comparable with individual psychological or psychoeducational interventions. This broader perspective is relevant because students’ responses to academic demands are shaped not only by individual coping resources but also by social, educational, and contextual factors [27,28,29,30,31].

Previous reviews provide support for school-based psychological and psychoeducational interventions, although their scope has generally extended beyond academic stress itself. Hikmat et al. [32] reported favourable findings for school-based psychological interventions targeting adolescent mental health, many of which incorporated cognitive-behavioural components. Similarly, López-Villegas and Sánchez-Sandoval [33] found that psychoeducational programmes implemented in school settings could improve mental health knowledge and promote relevant psychological and social skills. These findings support the value of school-based intervention, but they do not establish which approaches are most relevant specifically to academic stress or to students’ responses to academic demands. Other evidence has highlighted the importance of the educational context itself. Román Rentería and Llerena Companioni [34] reported associations between school demands and students’ emotional well-being, while also identifying family and school relationships as relevant to coping with academic stress. In a review focused on test anxiety, Jagiello et al. [14] reported favourable findings for cognitive-behavioural interventions, particularly for test anxiety, with more modest effects on general stress.

Despite this growing literature, the available evidence remains fragmented. Previous reviews have generally focused on broader school-based mental health interventions, particular psychological approaches, or specific outcomes such as test anxiety [14,32,33]. Jagiello et al. [14] specifically reviewed interventions for academic stress in high-school students and provided an important synthesis of this field. The present review extends this perspective in three specific respects: it examines a broader range of approaches addressing academic stress or academic demands, including socio-emotional and life-skills programmes, cognitive-behavioural and rational-emotive interventions, mindfulness- and acceptance-based approaches, stress-management and mindset interventions, and organisational approaches aimed at modifying academic burden; it incorporates a more recent evidence base, including studies published through August 2026; and it explicitly distinguishes interventions reporting direct measures of academic or school-related stress from those reporting related psychological or educational outcomes. This distinction is important because favourable changes in test anxiety, burnout, psychological distress, resilience, or self-efficacy cannot be assumed to represent reductions in academic stress itself. By examining intervention content, intervention level, and delivery alongside this distinction in outcomes, the present review provides an updated synthesis of a heterogeneous and developing evidence base.

Against this background, the present systematic review synthesises evidence on interventions addressing academic stress and academic demands among secondary school students. Specifically, it examines the intervention approaches represented in the literature, their principal components and delivery modalities, and the outcomes reported, while distinguishing direct measures of academic or school-related stress from related psychological and educational outcomes. The review also considers the methodological characteristics of the available evidence in order to identify current trends, limitations, and areas requiring further investigation.

Accordingly, the review addressed four research questions: (1) What interventions have been implemented to prevent or reduce academic stress or address academic demands among secondary school students? (2) What direct academic stress and related psychological or educational outcomes have been reported, and what evidence is available regarding intervention effectiveness? (3) What intervention approaches and delivery modalities are represented in the literature? (4) What methodological trends, limitations, and gaps can be identified in the available evidence?

## 2. Materials and Methods

This systematic review was conducted and reported in accordance with the PRISMA 2020 guidelines [35]. The database searches were completed on 12 August 2026. The review protocol was registered in the International Prospective Register of Systematic Reviews (PROSPERO; registration number CRD420261476775) on the same date, after completion of the database searches and before title and abstract screening, full-text eligibility assessment, and data extraction commenced.

### 2.1. Search Strategy

#### 2.1.1. Study Details

A systematic literature search was conducted across four databases: Web of Science, Scopus, PsycINFO, and PubMed. The search terms were structured around four key concepts: (1) academic stress; (2) the target population; (3) type of intervention; and (4) mental health or well-being. Boolean operators were used to combine the search terms, and the search strategy was adapted to the syntax and search functionalities of each database. Table 1 presents the search queries used in each database. These queries represent the search information retained for the review. Additional details on the exact field tags, interface-specific settings, and filters applied were not systematically retained and therefore cannot be reconstructed reliably. To avoid introducing retrospective assumptions, only the documented search information is reported. The Scopus strategy was more restrictive than those used in Web of Science, PsycINFO, and PubMed, particularly because it required “academic stress”, “adolescent”, “secondary school”, and either “stress management” or “mindfulness”. This difference may have reduced the sensitivity of the Scopus search and is therefore considered when interpreting the completeness of study identification. The mental health or well-being search block was used to retain a focus on interventions examining psychological or well-being outcomes in relation to academic stress and academic demands. However, requiring these terms may have reduced search sensitivity by excluding potentially relevant studies that focused specifically on academic stress without referring explicitly to mental health or well-being in the searchable fields. This possibility is acknowledged as a limitation of the search strategy. The database searches were completed on 12 August 2026. This date refers to the completion of the literature search and should not be interpreted as the date on which the systematic review was completed.

#### 2.1.2. Inclusion and Exclusion Criteria

The eligibility criteria were defined according to the PICOS framework [35] and documented in the registered protocol. The eligibility criteria specified in the registered protocol are summarised in Table 2. Deviations identified during the study-selection process are described separately below.

According to the registered eligibility criteria, studies were eligible if they: (1) were published in English or Spanish between 2015 and 2026 in peer-reviewed journals; (2) reported quantitative, qualitative, or mixed-methods empirical data; (3) included students in secondary education, generally aged 12–19 years; (4) examined an intervention, programme, or organisational or policy change aimed at preventing or reducing academic or school-related stress, helping students cope with academic demands, or reducing academic burden; and (5) assessed academic or school-related stress. Deviations from the population and outcome criteria that occurred during study selection are described below. Eligibility under the intervention criterion was considered independently from eligibility under the outcome criterion. Thus, the presence of a related psychological or educational outcome alone was not sufficient for inclusion; the intervention, programme, organisational change, or policy also had to address academic or school-related stress, coping with academic demands, or academic burden, as specified in the eligibility criteria. Reviews, theoretical papers, book chapters, and thesis chapters were excluded. Studies were also excluded if: (a) the intervention, programme, or organisational or policy change and the outcomes examined were unrelated to academic demands or school functioning; (b) the study population did not include secondary school students; (c) no relevant intervention, programme, or organisational or policy change was examined; or (d) the study was not sufficiently aligned with the review question or the full text was unavailable.

Two deviations from the eligibility criteria specified in the registered PROSPERO protocol occurred during the study-selection process. First, three studies with mixed-age or mixed educational-stage samples were retained because they included secondary school students and addressed interventions or contextual changes relevant to academic demands. Sellakumar [36] included students in Grades 6–12, aged 11–17 years, whereas the two studies evaluating the “Double Reduction” policy [37,38] included both primary and secondary school students. For the latter two studies, outcomes were not reported separately by educational stage and could therefore not be extracted specifically for secondary school students. Their findings were consequently treated as indirect or partially applicable evidence for secondary education rather than as fully representative of this population. Second, during full-text eligibility assessment, it became apparent that some studies evaluated interventions, programmes, or organisational or policy changes explicitly addressing academic demands but reported outcomes closely related to academic stress, such as test anxiety, school burnout, psychological distress, resilience, or self-efficacy, rather than measuring academic or school-related stress directly. These studies were retained because excluding them solely on the basis of the outcome measure would have omitted evidence on interventions specifically designed to address academic demands and students’ responses to them. This decision represented a deviation from the registered outcome criterion and is therefore reported explicitly. Once this decision was made, the revised outcome criterion was applied consistently to all studies undergoing full-text eligibility assessment, irrespective of the direction or statistical significance of their findings. The available review records do not allow us to establish whether the decision to broaden the outcome criterion preceded examination of all outcome data reported in potentially eligible full-text studies, and we therefore do not make this claim. Importantly, these related outcomes were not considered equivalent to academic stress. Studies directly assessing academic or school-related stress were treated as the primary, protocol-concordant outcome evidence, whereas studies retained under the expanded outcome criterion were treated as a secondary extension concerning related psychological or educational outcomes. This distinction was maintained throughout the synthesis and interpretation.

For the purposes of the synthesis, outcomes were classified into two groups: (1) direct academic-stress outcomes, when studies explicitly measured academic or school-related stress; and (2) related psychological or educational outcomes, including test anxiety, school burnout, psychological distress, depressive or anxiety symptoms, resilience, self-efficacy, academic engagement, and well-being, when these were examined in the context of interventions addressing academic demands. This distinction was maintained throughout data extraction, synthesis, and interpretation to avoid treating related outcomes as equivalent measures of academic stress.

#### 2.1.3. Selection of Studies

Using Rayyan.ai, the two reviewers managed the bibliographic records and identified and removed duplicate records. A total of 263 records were identified in Web of Science (WOS), 636 in Scopus, 179 in PsycINFO, and 203 in PubMed, yielding 1281 records. After duplicates were removed, 1068 studies remained for screening. The two reviewers independently screened the titles and abstracts of the retrieved records and subsequently assessed the full texts of potentially eligible studies according to the eligibility criteria described above. Inter-reviewer agreement was high (κ = 0.88), indicating almost perfect agreement. The two reviewers agreed on the inclusion or exclusion decision for 48 of the 51 studies assessed in full text (94.1% agreement). The three discrepancies identified were resolved through discussion and consensus, and the studies concerned were ultimately excluded from the review.

**Figure 1:**
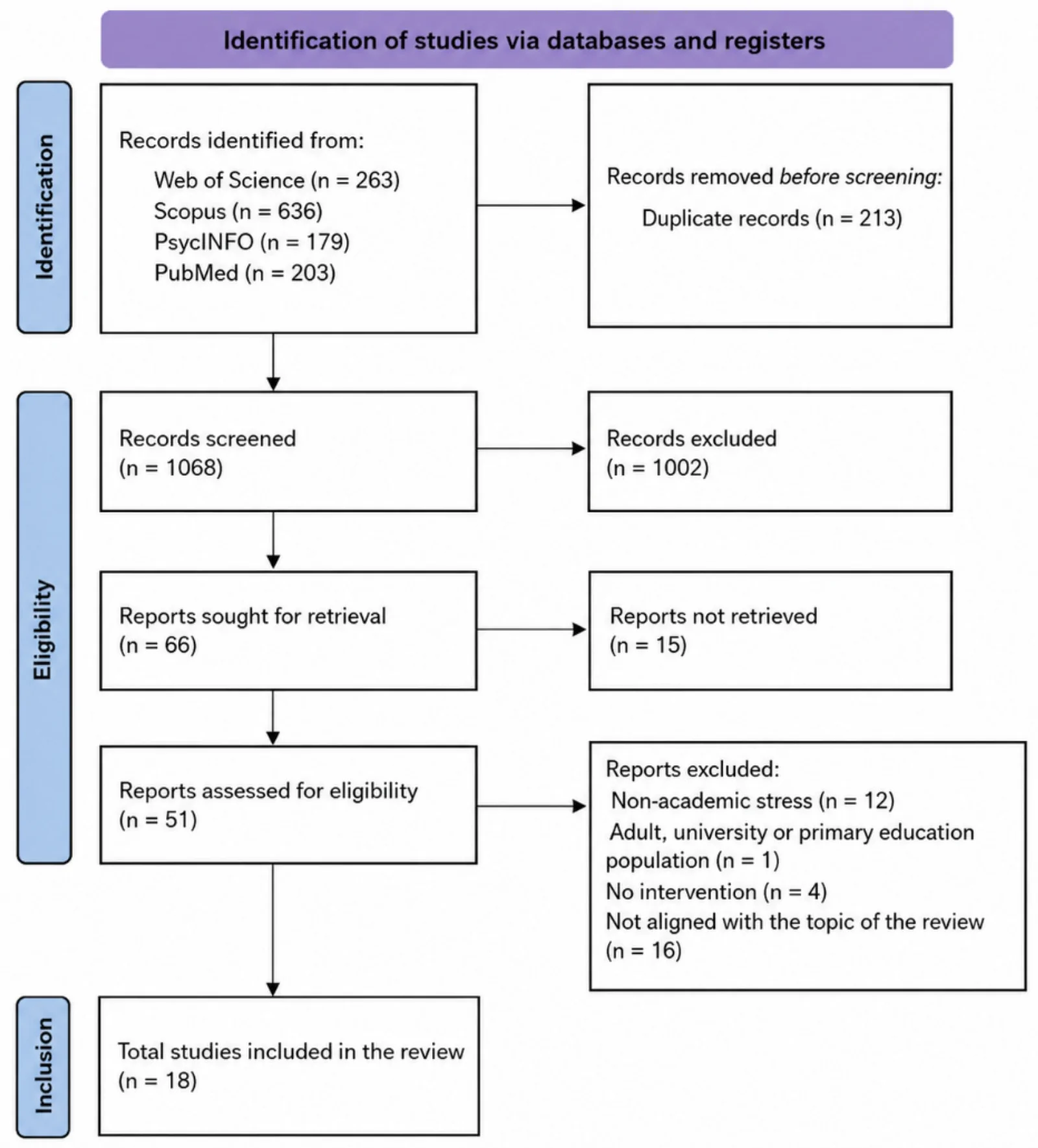
Flow Diagram of the Literature Search Results. Note. PRISMA 2020 flow diagram illustrating the process of study identification, selection, and inclusion. The different stages of the process are distinguished by colour: violet indicates record identification, while blue indicates the screening, eligibility, and inclusion stages.

Following full-text assessment, 33 studies were excluded because they did not meet the eligibility criteria applied in the review: 12 assessed forms of stress unrelated to academic or school demands, one did not include eligible secondary school participants, four did not examine an intervention or policy relevant to academic demands, and 16 did not meet one or more of the remaining eligibility criteria. Ultimately, 18 studies were retained and included in the systematic review (Figure 1).

#### 2.1.4. Data Extraction Process

Eighteen studies were manually coded by two reviewers independently (see Table S1). Any discrepancies in data extraction were resolved through discussion and consensus between the two reviewers. The data-extraction form was not formally piloted before its application to the included studies. The studies were coded according to the following characteristics: (1) study details (authors, year, and country); (2) participant characteristics, including educational stage, age, and sample size; (3) methodological design and allocation procedure, where applicable; (4) intervention characteristics and delivery modality; (5) outcome measures; (6) whether the study measured academic or school-related stress directly or assessed related psychological or educational outcomes; (7) main findings and reported effect estimates; and (8) methodological appraisal findings.

No data conversions, imputations, or transformations of summary statistics were performed. Effect estimates and statistical results were extracted and reported as presented in the original studies.

For the narrative synthesis, findings were organised according to intervention approach and outcome directness and were interpreted in relation to study design, the methodological limitations identified through the JBI or MMAT appraisal, and the consistency of findings across studies addressing comparable intervention approaches or outcomes. Greater interpretative weight was given to controlled experimental evidence when considering intervention effects, whereas uncontrolled, qualitative, and observational evidence was interpreted more cautiously in relation to perceived benefits, feasibility, temporal changes, or associations. Direct measures of academic or school-related stress were considered separately from related psychological or educational outcomes. No formal weighting score or certainty-of-evidence rating was assigned.

### 2.2. Methodological Appraisal

The included studies were methodologically appraised using the Joanna Briggs Institute (JBI) critical appraisal tools [39,40,41,42], selected according to study design, and the Mixed Methods Appraisal Tool (MMAT) [43] for mixed-methods studies.

The assessment considered the appropriateness of the methodological design, sample selection, presence of comparison groups where applicable, description of the intervention, validity and reliability of the instruments used, quality of data analysis, consistency between the results and conclusions, and potential sources of bias. Table 3 summarises the main methodological limitations identified for each study rather than a complete presentation of the appraisal results; item- or domain-level ratings were not retained for supplementary reporting.

For the two studies evaluating the ‘Double Reduction’ policy [37,38], the JBI Analytical Cross-Sectional Studies checklist was used because both studies were primarily structured as repeated cross-sectional surveys, although they also included nested longitudinal subsamples of students participating in both survey waves. The appraisal tool was selected according to the overarching observational survey design and the principal analyses evaluated in the review. The presence of the nested longitudinal component was nevertheless considered when interpreting the methodological strengths and limitations of these studies. All assessments were conducted independently by two reviewers. Two disagreements arose during this process and were resolved through discussion and consensus between the reviewers. Inter-reviewer agreement for the methodological appraisal was not formally quantified using an agreement statistic.

No formal assessment of reporting bias or publication bias was conducted because the review used a narrative synthesis and the included studies were highly heterogeneous in design, interventions, outcomes, and effect-size metrics, precluding meaningful quantitative assessment of small-study or publication-bias effects.

The certainty of the overall body of evidence was not formally assessed using a framework such as GRADE. Methodological appraisal was conducted at the individual-study level using the appropriate JBI or MMAT tool, and the identified strengths and limitations were considered when interpreting the narrative synthesis.

Methodological appraisal identified substantial variation in the strengths and limitations of the included studies. Recurrent limitations included small or context-specific samples, reliance on self-reported outcomes, absence of comparison groups in some studies, limited follow-up periods, and constraints related to allocation procedures and observational study designs. These issues were particularly relevant when interpreting findings from non-randomised and observational studies, for which causal attribution was more limited. The appraisal findings were therefore considered alongside study design, sample characteristics, and outcome measures when interpreting the evidence from individual studies and the overall narrative synthesis.

## 3. Results

The results are organised into thematic sections aligned with the research questions of the review, covering study selection and the characteristics of the included evidence, intervention approaches and delivery modalities, and intervention findings.

### 3.1. Study Selection

A total of 18 studies met the inclusion criteria established for the systematic review. The studies were published between 2015 and 2026 and were conducted across 10 countries. Six studies were published in 2024 [38,47,50,51,57,58], representing the largest number identified in a single year, while six of the 18 studies (33.3%) were conducted in China.

The included studies showed considerable methodological heterogeneity. Eight randomised or cluster-randomised controlled trials were identified [36,47,48,49,51,52,53,57], together with two non-randomised controlled or quasi-experimental studies [56,58], three mixed-methods studies [44,50,54], two qualitative studies [45,55], one implementation study [46], and two repeated cross-sectional studies with nested longitudinal subsamples [37,38]. Most intervention studies included pre-test and post-test assessments, whereas follow-up assessments were reported less consistently.

The included studies focused primarily on secondary school students, although a small number included participants outside the 12–19-year reference range or mixed educational-stage samples, as described in the protocol deviations reported in the Methods section. The two studies evaluating the “Double Reduction” policy [37,38] included both primary and secondary school students, while Sellakumar [36] included students in Grades 6–12, aged 11–17 years. Sample sizes varied considerably, from 28 participants in the smallest intervention study to 101,976 participants in the largest cross-sectional survey. In the two “Double Reduction” studies [37,38], the nested longitudinal subsamples comprised 28,398 and 36,637 students, respectively. Several studies focused on specific student groups, including students with learning difficulties, high academic stress or test anxiety, low academic achievement, or school burnout (see Table S1).

### 3.2. Intervention Approaches and Delivery Modalities

The identified interventions differed considerably in their theoretical orientation and components. For the purposes of synthesis, they were grouped according to their predominant intervention approach into five categories (Table 4): (1) socio-emotional and life-skills programmes (*n* = 4); (2) cognitive-behavioural and rational-emotive interventions (*n* = 5); (3) mindfulness- and acceptance-based interventions *(n* = 4); (4) stress-management and mindset interventions (*n* = 3); and (5) organisational approaches (*n* = 2). These categories were developed as an analytical framework for organising the heterogeneous evidence base rather than as mutually exclusive intervention types. Classification was based on the theoretical orientation explicitly described by the original authors and on the predominant intervention components reported in each study. The two reviewers independently classified the included interventions, and any discrepancies were resolved through discussion and consensus. When programmes incorporated components associated with more than one approach, they were assigned to the category that best reflected their predominant theoretical orientation and intervention content. Because this process necessarily involved some interpretative judgement, the categories should be understood as descriptive groupings for the purposes of narrative synthesis rather than as definitive classifications. Delivery modality was considered separately.

#### 3.2.1. Socio-Emotional and Life Skills Programmes

Four studies evaluated interventions focused primarily on socio-emotional competencies and life skills [44,45,50,55]. The TRY Gym programme addressed emotional, social, motivational, cognitive, and self-awareness competencies [44]. The ROBUST programme, examined in two studies, focused on interpersonal and relationship skills, problem-solving, emotional regulation, growth mindset, and mindfulness [45,55]. Nadaf et al. [50] evaluated a life-skills training programme that included self-awareness, emotional regulation, problem-solving, and effective communication. Although these programmes differed in structure and duration, they shared an emphasis on developing personal and interpersonal skills to help students cope with academic demands.

#### 3.2.2. Cognitive-Behavioural and Rational-Emotive Interventions

Five studies evaluated interventions based predominantly on Cognitive-Behavioural Therapy (CBT) or Rational Emotive Behaviour Therapy (REBT) approaches [46,48,51,54,57]. The Study Without Stress (SWOS) programme included psychoeducation, goal setting, time management, cognitive restructuring, problem-solving, relaxation, management of perfectionism and procrastination, and examination preparation [46,48]. The intervention evaluated by Omeke et al. [51] was based on REBT and included cognitive restructuring, reframing, time management, and problem-solving. Ulaş and Seçer [54] used a CBT-based psychoeducational programme to address school burnout, while the PASTEL programme combined CBT with study skills, including education about test anxiety, self-monitoring, cognitive restructuring, problem-solving, exposure, and organisational strategies [57].

#### 3.2.3. Mindfulness and Acceptance-Based Interventions

Four studies involved mindfulness- or acceptance-based interventions [47,52,53,58]. The Mindfulness in Schools Programme included mindfulness and breathing practices delivered in the classroom [47]. The AcAdeMiC programme addressed test and examination anxiety through acceptance, mindfulness, compassion, and psychoeducation [52]. Youth COMPASS was based on Acceptance and Commitment Therapy and included acceptance skills, mindfulness, self-compassion, and adaptation [53]. Yue et al. [58] evaluated a brief meditation intervention that included breathing meditation, mindful physical relaxation, awareness of emotional states, and consolidation of meditation techniques.

#### 3.2.4. Stress-Management and Mindset Interventions

Three studies evaluated interventions focused on stress-management or mindset strategies [36,49,56]. Melhe et al. [49] examined a training programme based on positive thinking in students with low academic achievement. Sellakumar [36] used slow deep-breathing exercises to reduce anxiety among school students, while Wang et al. [56] evaluated a brief classroom course aimed at promoting a stress-is-enhancing mindset by helping students recognise the potential benefits of stress.

#### 3.2.5. Organisational Approaches

Two studies examined organisational changes associated with the “Double Reduction” policy implemented in China [37,38]. The policy aimed to reduce students’ academic burden through measures such as limiting homework and extracurricular tutoring. The studies also considered changes in extracurricular activities, physical activity, family time, academic stress, and sleep.

#### 3.2.6. Delivery Modalities

Most interventions were delivered face-to-face, generally within the school setting. Pires et al. [52] delivered the intervention online through individual sessions via Zoom. The Youth COMPASS programme evaluated by Puolakanaho et al. [53] was delivered via the Internet and mobile devices, with one intervention condition also including face-to-face meetings. The two studies evaluating the “Double Reduction” policy involved organisational changes implemented within schools [37,38].

### 3.3. Intervention Findings

Intervention findings were synthesised descriptively, distinguishing between direct measures of academic or school-related stress and related psychological or educational outcomes. Of the 18 included studies, four directly assessed academic or school-related stress as an intervention outcome [49,50,51,53] and therefore constituted the primary outcome evidence most closely aligned with the registered outcome criterion. The remaining 14 studies [36,37,38,44,45,46,47,48,52,54,55,56,57,58] did not directly assess academic or school-related stress as an intervention outcome and were retained under the post-registration expansion of the outcome criterion. These studies are presented as a secondary extension of the evidence base concerning related psychological or educational outcomes. This section focuses on patterns within the intervention approaches, while Section 3.4 summarises related psychological and educational outcomes across approaches to avoid duplicating detailed study-level descriptions. Statistical significance and effect estimates were reported as presented in the original studies, while qualitative findings were synthesised according to participants’ reported experiences. Given the heterogeneity of study designs, outcome measures, and effect-size metrics, findings were interpreted within individual studies rather than directly compared using a common quantitative threshold.

Among socio-emotional and life-skills interventions, direct evidence for academic stress was limited to one mixed-methods study without a comparison group, which reported a reduction following life-skills training [50]. The remaining evidence in this intervention category was derived from mixed-methods and qualitative studies and primarily concerned related socio-emotional, well-being, or coping outcomes, including students’ perceived ability to manage academic demands [44,45,55].

Within CBT- and REBT-based interventions, only Omeke et al. [51] directly assessed academic stress and reported a reduction following the intervention. Jagiello et al. [46] and Lowe and Wuthrich [48] reported changes in general stress or psychological distress using the DASS-21; these outcomes were therefore treated as related psychological outcomes rather than as direct measures of academic or school-related stress. Other studies in this category reported changes in related outcomes, including school burnout and test anxiety [54,57].

Among mindfulness- and acceptance-based interventions, only Puolakanaho et al. [53] assessed general and school-related stress directly, reporting a reduction in general stress, whereas the effect on school-related stress was less clearly established. The remaining studies primarily reported changes in related outcomes, including learning burnout, test anxiety, mindfulness, psychological resilience, self-compassion, and self-efficacy [47,52,58]. These findings should not be interpreted as direct evidence of reductions in academic stress.

Findings from the three stress-management and mindset interventions varied according to the outcome assessed. Melhe et al. [49] reported a reduction in academic stress together with an improvement in psychological hardiness following positive-thinking training. Sellakumar [36] did not assess academic stress directly but reported significant reductions in state, trait, and overall anxiety following slow deep-breathing exercises, with significant time × group interactions (ηp^2^ = 0.464, 0.362, and 0.567, respectively). Wang et al. [56] found a significant increase in stress-is-enhancing mindset, whereas no significant overall reduction in perceived stress was observed (*p* = 0.715).

The two observational studies examining the “Double Reduction” policy primarily assessed depressive and anxiety symptoms rather than academic stress as an intervention outcome. Wang et al. [37] reported decreases in depression and anxiety after policy implementation, with reduced academic stress, lower homework burden, more extracurricular activities, more time with parents, and longer sleep duration associated with a lower likelihood of mental health problems. Wang et al. [38] similarly reported decreases in depressive and anxiety symptoms one year after policy implementation and identified reduced academic stress and other policy-related factors as correlates of better mental health outcomes. As both studies used observational pre–post designs without an unexposed comparison group, these findings indicate temporal changes and associations rather than causal effects of the policy.

The evidence concerning organisational approaches should be interpreted particularly cautiously because the two studies evaluating the “Double Reduction” policy [37,38] included mixed primary and secondary school populations and did not report outcomes separately by educational stage. Consequently, the findings cannot be attributed specifically to secondary school students. If these mixed-stage studies were excluded, the organisational-level approach would no longer be represented in the present review, whereas the evidence concerning the individual-level intervention categories would remain unchanged. The organisational findings should therefore be regarded as contextual and preliminary rather than as evidence directly generalisable to secondary education.

### 3.4. Associated Psychological and Educational Outcomes

#### 3.4.1. Well-Being and Psychological Distress

Several studies reported outcomes related to mental well-being and psychological distress, with favourable changes reported for well-being or distress in the TRY Gym and SWOS programmes and in a randomised CBT-based intervention [44,46,48]. These outcomes were not consistently assessed across studies and should be distinguished from direct measures of academic or school-related stress.

#### 3.4.2. Depression

Five studies reported depressive symptoms [37,38,46,48,57]. Findings varied across intervention approaches: no significant reductions were reported in two studies [46,48], whereas decreases were observed following the PASTEL programme [57] and after implementation of the “Double Reduction” policy [37,38]. The latter findings were derived from uncontrolled observational studies and therefore represent temporal changes rather than evidence of causal policy effects.

#### 3.4.3. Anxiety

Eight studies reported anxiety-related outcomes [36,37,38,46,48,52,57,58]. Findings were mixed for general anxiety [36,46,48,57], whereas reductions in test anxiety were reported across several intervention approaches [52,57,58]. The “Double Reduction” studies also reported decreases in anxiety symptoms following policy implementation [37,38], although their observational designs preclude causal attribution. Anxiety-related outcomes were treated as related outcomes rather than as direct measures of academic stress.

#### 3.4.4. Resilience and Related Personal Resources

Several studies reported changes in personal resources potentially relevant to coping with academic demands, including resilience, psychological hardiness, academic buoyancy, self-compassion, and mindfulness [44,47,49,50,52,53,58]. Favourable changes were reported for several of these constructs, although findings were not uniform across all outcomes. Because these constructs represent distinct psychological resources, they were not interpreted as equivalent to one another or as direct measures of academic stress.

#### 3.4.5. Self-Efficacy

Four studies assessed self-efficacy [44,48,57,58], with mixed findings across interventions. Improvements were reported in some studies [48,58], whereas others found no significant changes in specific self-efficacy outcomes [44,57]. Self-efficacy was treated as a related psychological or educational outcome rather than as a direct measure of academic stress.

#### 3.4.6. Academic and Social Outcomes

Several studies reported academic or social outcomes, including academic engagement, academic performance, school adjustment, interpersonal skills, sense of belonging, communication, and social competence [44,45,50,51]. Favourable changes were reported for several of these outcomes, although school adjustment did not significantly improve in one study [51]. These findings describe related educational and social outcomes and were not interpreted as direct evidence of reductions in academic stress.

## 4. Discussion

### 4.1. Main Findings

This review identified a heterogeneous body of evidence on interventions addressing academic stress and responses to academic demands among secondary school students. Importantly, direct intervention evidence aligned with the originally registered outcome criterion was relatively limited: only four of the 18 included studies directly assessed academic or school-related stress as an intervention outcome [49,50,51,53]. The remaining 14 studies were retained following the post-registration expansion of the outcome criterion and assessed related psychological or educational outcomes rather than academic or school-related stress directly. These studies broaden the evidence concerning interventions addressing academic demands, but they should be regarded as a secondary extension of the evidence base rather than as direct evidence addressing the original outcome criterion. Related outcomes included test anxiety, school burnout, psychological distress, resilience, and self-efficacy, which should not be interpreted as interchangeable indicators of academic stress. Consequently, favourable changes in these outcomes cannot be assumed to represent reductions in academic stress itself. The diversity of study designs, outcome measures, intervention components, and effect-size metrics further limits direct comparison between studies and does not support identifying any one intervention approach as clearly superior.

The methodological appraisal also indicates that the findings should be weighted according to study design. Evidence from randomised controlled trials provides a stronger basis for attributing observed changes to the interventions, although several trials were limited by small samples, short follow-up periods, or other design-specific constraints. In contrast, findings from uncontrolled, qualitative, and observational studies are informative regarding feasibility, acceptability, perceived benefits, and associations, but provide more limited evidence of intervention effects. This distinction is particularly relevant to the organisational-level evidence, which was derived from observational studies without an unexposed comparison group. Accordingly, favourable findings across intervention categories should not be interpreted as providing equivalent levels of evidence.

The findings show some convergence with previous reviews, although they should be interpreted cautiously given the small number of studies within each intervention category and differences in methodological design and limitations. Hikmat et al. [32] found that cognitive-behavioural components were common in psychological interventions implemented in school settings, while Jagiello et al. [14] reported favourable findings for cognitive-behavioural programmes, particularly in relation to test anxiety and, to a lesser extent, general stress. In the present review, CBT- and REBT-based interventions reported changes in stress, distress, academic stress, school burnout, and test anxiety, but findings were not consistent across all outcomes and were derived from studies with differing methodological strengths. Mindfulness- and acceptance-based interventions also reported favourable changes in several related outcomes, including test anxiety, learning burnout, mindfulness, psychological resilience, self-compassion, and academic buoyancy. These changes describe distinct psychological or educational outcomes and should not be interpreted as reductions in academic stress unless academic or school-related stress was directly assessed. Direct evidence for academic or school-related stress within this category was limited and less consistent. Socio-emotional and life-skills interventions similarly reported favourable findings for several related outcomes, but direct evidence for academic stress was limited to one mixed-methods study without a comparison group [50]. Accordingly, findings across these intervention categories should be regarded as suggestive rather than as evidence of consistent effectiveness for reducing academic stress.

Several recurring components were identified across interventions with different theoretical orientations. Problem-solving and emotional regulation were present in socio-emotional and life-skills programmes, while problem-solving, cognitive restructuring, and strategies for managing academic demands were recurrent in CBT- and REBT-based interventions. Mindfulness- and acceptance-based programmes placed greater emphasis on awareness, acceptance, compassion, and emotional regulation. This overlap indicates that some components extended across the intervention categories used in this review. These shared components represent plausible pathways through which interventions may influence students’ responses to academic demands. For example, cognitive restructuring and problem-solving may support the appraisal and management of academic demands, whereas mindfulness, acceptance, and emotional-regulation strategies may influence responses to stress-related thoughts and emotions. However, these mechanisms were rarely examined directly in the included studies. Because most programmes combined multiple components and mediation or component-specific analyses were uncommon, the present review cannot determine the independent effectiveness or contribution of individual intervention components.

This is particularly relevant given the academic demands identified in previous research. Workload, time pressure, and assessment-related demands have been identified as common sources of academic stress among students [5,10,19,20,22,31]. The interventions included in the present review addressed these demands in different ways, including problem-solving, time management, examination preparation, emotional regulation, mindfulness, and strategies for modifying students’ responses to stress. Social and interpersonal outcomes were also reported in several of the socio-emotional and life-skills programmes. The TRY Gym programme produced significant improvements in social competence [44], while students participating in ROBUST described improvements in interpersonal skills and sense of belonging, together with greater academic engagement [45]. Nadaf et al. [50] also reported improvements in communication and other life skills. These findings are consistent with the interpersonal emphasis of these programmes, although the available evidence does not establish whether changes in social competence contribute directly to reductions in academic stress.

Delivery modality varied less than intervention content, as most interventions were implemented face-to-face and generally within school settings. Pires et al. [52] delivered the AcAdeMiC programme online through individual Zoom sessions, whereas Youth COMPASS combined Internet- and mobile-based delivery with face-to-face meetings in one intervention condition [53]. The online AcAdeMiC programme reduced test anxiety and increased self-compassion, while Youth COMPASS reduced general stress and increased academic buoyancy, although changes in school-related stress were not clearly established. Given the small number of studies using digital or blended delivery and the differences in their intervention content and designs, no conclusions can be drawn about the comparative effectiveness of digital and face-to-face modalities.

The geographical and educational contexts represented in the review also differed considerably. Interventions were implemented across different school systems and cultural settings, while six of the 18 studies were conducted in China. Organisational approaches were particularly context-dependent, as the “Double Reduction” policy reflects changes specific to the Chinese educational system. Student-focused interventions may likewise be influenced by differences in school organisation, academic expectations, assessment practices, and available support structures. However, the small number of studies within individual countries and the heterogeneity of interventions and outcomes do not permit meaningful cross-country comparisons. Context should therefore be considered when interpreting the transferability of the findings rather than as an independent explanation for differences in intervention effects.

### 4.2. Implications for Educational Practice

The findings suggest that academic stress may be addressed through existing school guidance and support structures, in addition to interventions focused on individual students. Several interventions were delivered during regular school activities by teachers, tutors, or school counsellors. ROBUST was implemented by teachers during school hours, SWOS was delivered by tutors, the Mindfulness in Schools Programme was delivered by a teacher in the classroom, and the life-skills programme evaluated by Nadaf et al. [50] involved teachers and school counsellors [45,46,47,50,55]. In the Spanish educational context, Tutorial Action Plans (PAT) provide an example of an existing school-based structure through which socio-emotional learning, coping strategies, mindfulness practices, or stress-management activities might be integrated into routine educational practice. This is presented as a context-specific example rather than as a recommendation supported by the international evidence synthesised in this review.

The findings also suggest that prevention should not be limited to interventions aimed at developing individual coping skills. The two studies examining the “Double Reduction” policy found that reduced homework burden, greater participation in extracurricular activities, more family time, lower academic stress, and adequate sleep were associated with a lower likelihood of mental health problems [37,38]. These results highlight the importance of considering workload and school organisation alongside student-focused interventions. However, both studies used observational designs without control groups and were conducted by the same research group using related survey waves, with potentially overlapping participant samples. The reported associations should therefore not be interpreted as evidence that these organisational changes caused the reductions in depressive or anxiety symptoms, nor should the two studies be regarded as fully independent evidence.

Intervention format also needs to be adapted to the educational context and students’ needs. The studies included classroom lessons, workshops, individual sessions, youth-led activities, and digital or blended delivery, without providing sufficient evidence to establish that one format is preferable. Participatory elements were incorporated in programmes such as TRY Gym and ROBUST, but their specific contribution to engagement or intervention outcomes was not assessed independently [44,45].

## 5. Limitations

Several limitations should be considered when interpreting the findings of this review. First, the evidence base was relatively small, comprising 18 studies, and the included studies showed considerable heterogeneity in methodological design, intervention characteristics, populations, outcome measures, and effect-size metrics. This limited direct comparisons between interventions and did not allow one approach to be identified as more effective than the others. For the same reason, a meta-analysis was not considered appropriate, and the findings were synthesised narratively. Some studies also involved small or specific samples, lacked control groups, or used qualitative or observational designs, limiting the strength of causal conclusions for some of the interventions examined. In addition, the two studies examining the “Double Reduction” policy were conducted by the same research group using related survey waves and potentially overlapping participant samples [37,38]. Their findings should therefore not be interpreted as two fully independent replications of the effects associated with the policy.

Two deviations from the eligibility criteria specified in the registered protocol were identified. A small number of studies included participants outside the 12–19-year reference range or mixed primary and secondary school samples. More importantly, the registered outcome criterion was expanded during full-text eligibility assessment to include studies assessing psychological or educational outcomes related to academic stress rather than academic or school-related stress directly. This post-registration change represents a methodological limitation because it broadened the scope of the review beyond the outcome criterion specified in the protocol and increased the heterogeneity of the evidence base. As described in the Methods, once this decision was made, the revised outcome criterion was applied consistently to all studies undergoing full-text eligibility assessment, irrespective of the direction or statistical significance of their findings. Nevertheless, the deviation should be considered when interpreting the scope and conclusions of the review. Related outcomes such as test anxiety, school burnout, depression, resilience, and self-efficacy were therefore analysed as distinct constructs rather than as equivalent measures of academic stress.

The geographical distribution of the studies also limits the generalisability of the findings. Six of the 18 studies were conducted in China, while several other countries were represented by only one study. Differences in educational, cultural, and organisational contexts should therefore be considered when interpreting the findings. Digital and blended interventions were uncommon, and only two studies examined organisational changes, limiting the conclusions that can be drawn about these approaches. Few studies provided long-term follow-up data, so it remains unclear whether the observed effects persist over time. No interventions involving families directly in programme delivery were identified, despite the relevance of the family context during adolescence.

The search was also restricted to four databases and to studies published in English or Spanish. In addition, the search strategies were not equivalent across databases, and the Scopus strategy was more restrictive than those used in Web of Science, PsycINFO, and PubMed. In addition, because exact database field tags, interface-specific settings, and filters were not systematically retained, the search strategies cannot be fully reconstructed, which limits the reproducibility of the search. This may have reduced search sensitivity and affected the completeness of study identification. The requirement for mental health or well-being terms may also have excluded potentially relevant studies focused specifically on academic stress. Importantly, the database searches were designed around the original academic-stress-focused scope and were not rerun after the outcome criterion was expanded. Consequently, the original search strategy cannot be assumed to have comprehensively identified the broader literature on the related psychological and educational outcomes subsequently considered eligible. Evidence retained under the expanded outcome criterion should therefore be interpreted as a secondary extension rather than as a comprehensive systematic representation of this broader evidence base. Relevant studies indexed elsewhere, published in other languages, or not captured by the database-specific search strategies may therefore have been missed.

Because item- or domain-level JBI and MMAT ratings were not retained, the methodological appraisal can only be reported as a summary of the principal limitations identified for each study, limiting the reproducibility and independent verification of the appraisal findings.

Finally, publication bias was not formally assessed, and the certainty of the overall body of evidence was not evaluated using a framework such as GRADE. These considerations further limit the strength of conclusions that can be drawn from the available evidence.

## 6. Conclusions

The available evidence suggests that several intervention approaches may be relevant to addressing academic stress and responses to academic demands among secondary school students. However, direct evidence aligned with the originally registered outcome criterion remains limited: academic or school-related stress was directly assessed as an intervention outcome in only four of the 18 included studies. The remaining 14 studies were retained following the post-registration expansion of the outcome criterion and constitute a broader, more heterogeneous body of evidence concerning related psychological or educational outcomes rather than direct academic-stress outcomes. Favourable findings were reported across socio-emotional and life-skills programmes, cognitive-behavioural and rational-emotive interventions, mindfulness- and acceptance-based interventions, and stress-management and mindset interventions, although these findings varied across studies and outcomes and should not be interpreted as consistent evidence of reductions in academic stress. Organisational changes aimed at reducing academic burden were also associated with changes in mental health indicators, but this evidence was limited to two observational studies without control groups.

Given the heterogeneity of study designs, outcome measures, intervention characteristics, and effect-size metrics, the available evidence does not support broad conclusions about overall intervention effectiveness or the comparative superiority of any intervention approach. Most interventions were delivered face-to-face, while evidence on digital and blended delivery remains limited. Further research should include well-controlled studies with longer follow-up periods, use more consistent measures of academic stress, and examine interventions across different educational and cultural contexts. When evaluating intervention effects, academic stress should also be distinguished from related psychological and educational outcomes.

## Figures and Tables

**Table 1:** Search queries used.

Database	Search Strategy
Web of Science	(“academic stress” OR “school stress”) AND (adolescent * OR teen * OR youth OR “secondary school” OR “high school”) AND (intervention OR prevention OR program *) AND (“mental health” OR wellbeing OR “well-being”)
Scopus	(“academic stress”) AND (adolescent *) AND (intervention OR prevention) AND (“secondary school”) AND (“stress management” OR mindfulness)
PsycINFO	(“academic stress” OR “school stress”) AND (adolescent * OR teen * OR youth OR “secondary school” OR “high school”) AND (intervention OR prevention OR program *) AND (“mental health” OR wellbeing OR “well-being”)
PubMed	(“academic stress” OR “school stress”) AND (adolescent * OR teen * OR youth OR “secondary school” OR “high school”) AND (intervention OR prevention OR program *) AND (“mental health” OR wellbeing OR “well-being”)

Note. * = truncation wildcard. The search blocks were combined using AND, while the terms within each block were combined using OR.

**Table 2:** PICOS Eligibility Criteria.

Criterion	Component	Inclusion Criteria	Exclusion Criteria
P	Population	Secondary school students, generally aged 12–19 years.	Primary-only, university, adult, or unrelated clinical populations.
I	Intervention	Interventions, programmes, or organisational or policy changes addressing academic/school-related stress, coping with academic demands, or a reduction in academic burden.	No intervention, programme, or organisational or policy change relevant to academic demands or school-related stress.
C	Comparison	Control group, comparison condition, pre–post assessment, or no comparator when consistent with the methodological design of the study.	Studies without a comparison group or baseline measurement when required by the methodological design.
O	Outcomes	Academic or school-related stress.	Outcomes unrelated to academic demands or school functioning.
S	Study Design	Original empirical studies: quantitative, qualitative, or mixed-methods studies.	Systematic reviews; theoretical papers; book chapters; undergraduate theses; editorials; letters to the editor.
Date	Publication period	2015–2026.	Publications prior to 2015.
Language	Language	English or Spanish.	Languages other than English or Spanish.

Note. The eligibility criteria shown in the table correspond to those specified in the registered protocol. Two deviations identified during study selection are described in detail below. These concerned the inclusion of a small number of mixed-age or mixed educational-stage samples and studies assessing psychological or educational outcomes related to academic stress in the context of interventions addressing academic demands. These related outcomes were not considered equivalent to academic stress and were analysed separately throughout the review. Table compiled by the authors in accordance with the guidelines set out by Page et al. [35].

**Table 3:** Summary of the Main Methodological Limitations Identified in the Included Studies.

Authors	Design	Tool	Main Limitations
Chung et al. (2026) [44]	Mixed methods	MMAT	Context-specific sample, which may limit the transferability of the findings to other educational settings.
Eriksen et al. (2025) [45]	Qualitative	JBI Qualitative	Findings are context-dependent and based on students’ self-reported experiences, which may limit their transferability to other educational settings.
Jagiello et al. (2022) [46]	Implementation study	JBI Quasi-experimental	Single-school implementation study without a comparison group, limiting causal attribution and generalisability of the findings.
Liu et al. (2024) [47]	Cluster-randomised controlled trial	JBI-RCT	Cluster-randomised design with a relatively small sample (N = 125); the context-specific sample may also limit the transferability of the findings.
Lowe and Wuthrich (2021) [48]	Randomised controlled trial	JBI-RCT	Small sample size (N = 56) and follow-up limited to three months.
Melhe et al. (2021) [49]	Randomised controlled trial	JBI-RCT	Very small, purposively selected sample (N = 28), restricted to male seventh-grade students with low academic achievement, limiting the generalisability of the findings.
Nadaf et al. (2024) [50]	Mixed methods	MMAT	Absence of a comparison group and purposive sampling of students with learning disabilities, limiting causal inference and the generalisability of the findings.
Omeke et al. (2024) [51]	Randomised controlled trial	JBI-RCT	Context-specific sample of students from rural schools, which may limit the generalisability of the findings to other educational settings.
Pires et al. (2026) [52]	Randomised pilot trial	JBI-RCT	Small pilot sample (N = 35), with missing data reducing the effective sample size for some repeated-measures analyses.
Puolakanaho et al. (2019) [53]	Randomised controlled trial	JBI-RCT	Intervention effects were small and differed between intention-to-treat and per-protocol analyses, warranting caution in interpreting efficacy.
Sellakumar (2015) [36]	Randomised controlled trial	JBI-RCT	Single-school sample and reliance on a self-reported measure of anxiety, which may limit the generalisability and objectivity of the findings.
Ulaş and Seçer (2022) [54]	Mixed methods	MMAT	Small experimental sample (N = 30), limiting the generalisability of the quantitative findings.
Vestad and Tharaldsen (2022) [55]	Qualitative	JBI Qualitative	Small qualitative sample (*n* = 26) drawn from a single Norwegian municipality, limiting the transferability of the findings to other educational contexts.
Wang et al. (2022) [37]	Repeated cross-sectional study with a nested longitudinal subsample	JBI Analytical Cross-sectional	Observational pre–post policy design without an unexposed comparison group, limiting causal attribution of changes to the “Double Reduction” policy.
Wang et al. (2024) [38]	Repeated cross-sectional study with a nested longitudinal subsample	JBI Analytical Cross-sectional	Observational pre–post policy design without an unexposed comparison group, limiting causal attribution of the observed changes to the “Double Reduction” policy.
Wang et al. (2026) [56]	Controlled intervention study	JBI Quasi-experimental	Single-session intervention conducted in one school, with a brief post-intervention assessment period, limiting conclusions about the durability and generalisability of the effects.
Yale-Soulière et al. (2024) [57]	Randomised controlled trial	JBI-RCT	Small sample size (N = 48), limiting statistical power and the generalisability of the findings.
Yue et al. (2024) [58]	Quasi-experimental	JBI Quasi-experimental	Class-level allocation without individual randomisation and recruitment from a single school, limiting control over potential group differences and the generalisability of the findings.

Note. RCT = Randomised Controlled Trial; MMAT = Mixed Methods Appraisal Tool.

**Table 4:** Summary of intervention approaches and delivery modalities.

Intervention Approach	Studies (*n*)	Main Components	Predominant Delivery Modality
Socio-emotional and life-skills programmes	4	Emotional regulation, interpersonal skills, problem-solving, self-awareness, communication, growth mindset	Face-to-face
Cognitive-behavioural and rational-emotive interventions	5	Psychoeducation, cognitive restructuring, problem-solving, time management, exposure, study skills	Face-to-face
Mindfulness- and acceptance-based interventions	4	Mindfulness, meditation, acceptance, compassion, breathing, emotional awareness	Face-to-face, online, or blended
Stress-management and mindset interventions	3	Positive thinking, slow deep breathing, stress mindset	Face-to-face
Organisational approaches	2	Reduction of homework and extracurricular tutoring, changes in academic burden and school organisation	School-level implementation

## Data Availability

No new data were created or analyzed in this study. Data sharing is not applicable to this article.

## Supplementary Materials

The following supporting information can be downloaded at: https://www.mdpi.com/article/10.3390/healthcare14193346/s1, Table S1: Characteristics of the Studies Included in the Systematic Review.

## Abbreviations

The following abbreviations are used in this manuscript:

AcAdeMiC	Acting with Acceptance, Mindfulness and Compassion to Cope with Test and Examination Anxiety
CBT	Cognitive-Behavioural Therapy
JBI	Joanna Briggs Institute
MMAT	Mixed Methods Appraisal Tool
PAT	Plan de Acción Tutorial (Tutorial Action Plan)
PROSPERO	International Prospective Register of Systematic Reviews
RCT	Randomised Controlled Trial
REBT	Rational Emotive Behaviour Therapy
SWOS	Study Without Stress
TRY	Transformative, Resilient, and Youth-Led
