# Supplementary material for: Addressing Academic Stress in Secondary Education: A Systematic Review of Intervention Approaches and Related Outcomes
Source: Healthcare (Basel). 2026 Oct 8;14(19):3346. doi: 10.3390/healthcare14193346 (PMC13664731; doi:10.3390/healthcare14193346)
Supplement: Supplementary file 1 [file healthcare-14-03346-s001.zip › healthcare-4579939-supplementary.pdf]

Table S1. Characteristics of the Studies Included in the Systematic Review

Study Identification	Sample Characteristics	Methodological Design	Intervention Characteristics	Basis for Intervention Eligibility	Variables Analysed	Outcome Classification	Main Results	Reported Effect Estimates and Statistical Results
Chung et al. (2026) [41] China	Secondary school students in Forms 3–5 (n = 80). Eight schools. Age range: 14–19 years; M = 16.05 years.	Mixed-methods design. No control group. Duration: one academic year. Three stages: training (8 sessions), individual practice (4 weeks), and implementation (at 6 months). Assessment at baseline and after training and implementation.	TRY Gym programme (Transformative, Resilient and Youth-led). Mental health training addressing academic stress. Focused on five competencies: emotional, social, motivational, cognitive, and self-awareness. Participants acted as youth mentors and were required to organise at least three mental health-related events. Delivery mode: face-to-face.	Academic-stress target	Dependent variables: social, cognitive, and emotional competence; resilience; and mental well-being. Measures: semi-structured qualitative interviews (n = 19); modified Chinese Positive Youth Development Scale (CPYDS); Revised Motivation Scale; Student Opinion Scale (SOS); Perceived Devaluation-Discrimination Scale; Warwick–Edinburgh Mental Well-Being Scale (WEMWBS); Self-Compassion Scale for Youth (SCS-Y); Strengths Use and Current Knowledge Scale; Attitudes Toward Seeking Mental Help Scale.	Related psychological/ educational outcome	Significant improvements in social skills, cognitive competence, emotional regulation, resilience, ability to cope with challenges, and mental well-being. Self-compassion, self-efficacy competence, and motivational competence showed no significant changes. Participants considered that the programme improved their understanding and awareness of mental health.	Social competence ($p = 0.001$, $r = 0.41$); cognitive competence ($p = 0.009$, $r = 0.33$); emotional regulation ($p = 0.024$, $r = 0.30$); mental well-being ($p = 0.022$, $r = 0.30$).
Eriksen et al. (2025) [42] Norway	Grade 9 secondary school students. 43 classes.	Qualitative design. No control group. Duration: September 2021 to April 2022. Twenty-five lessons lasting 45–60 min. Five thematic blocks, 5 h each.	ROBUST intervention. Social and Emotional Learning (SEL) covering five competencies: interpersonal skills, problem-solving, emotional regulation, growth mindset, and mindfulness. Face-to-face classroom delivery by teachers.	Academic-demand target	Dependent variable: academic engagement. Measures: three semi-structured interviews and a subsequent member check. Focus group (n = 56), conducted in September 2022. Thematic analysis using an abductive approach.	Related psychological/ educational outcome	Beneficial for coping with academic stress, improving problem-solving, regulating emotions, initiating schoolwork, persistence, developing more constructive emotional responses, recognising negative and positive thoughts, increasing confidence in the ability to overcome academic challenges, improving interpersonal skills, and strengthening sense of	Participants responded positively to several components of the intervention. The findings supported the acceptability of this SEL intervention among secondary school students.

							belonging and academic engagement.	
Jagiello et al. (2022) [43] Australia	Grade 11 secondary school students (n = 80). One school. Age range: 16–17 years.	Implementation study. Longitudinal. No control group. Duration: final school term, with the last session delivered during the first week of Grade 12. Pretest, posttest, and 3-month follow-up.	“Study Without Stress” (SWOS) programme based on CBT for school-related stress. Eight 30-min modules: psychoeducation, goal setting, links between thoughts and feelings, time management and relaxation, challenging thoughts, reducing perfectionism and procrastination, problem-solving, examination preparation, and relapse prevention. Face-to-face delivery at school by tutors. Five tutorial groups of 15–17 students.	Academic-stress target	Dependent variables: depression, anxiety, stress, and total distress. Measures: Depression Anxiety Stress Scales–Short Form (DASS-21); Mental Health Literacy Questionnaire (MHLQ); School Intervention Rating Form–Student Version (SIRF-S); School Intervention Rating Form–Facilitator Version (SIRF-F); School Intervention Rating Form–Other Teachers Version (SIRF-OT); Normalisation Measure Development Questionnaire (NoMAD).	Related psychological/ educational outcome	Maintenance of normal stress levels over time. Although stress increased significantly among students in the low-stress group, it remained within the normal range. Stress and distress decreased among students presenting high baseline levels. Reductions in depression and anxiety were not significant. A significant overall change was observed in students’ reported use of strategies.	significant reductions in stress among students with high distress ($t(113.92) = 3.60$, $p = 0.001$) and in total distress ($t(114.14) = 2.75$, $p = 0.020$). No significant reductions were observed for depression or anxiety (all $p > 0.05$).
Liu et al. (2024) [44] China	Students from 14 Grade 8 classes (n = 117). One school. Mean age: experimental group = 15.86 years; control group = 15.92 years.	Experimental cluster-randomised controlled trial. Control group (n = 60). Duration: 10 sessions, one 45-min session per week, plus 10–15 min of daily practice. Pretest and posttest assessments.	Mindfulness. Chinese version of the Mindfulness in Schools Programme (MiSP.b). Face-to-face classroom delivery by a teacher.	Academic-demand target	Dependent variables: academic burnout, dispositional mindfulness, and psychological resilience. Measures: Academic Burnout Inventory for Adolescent Students; Chinese version of the Five Facet Mindfulness Questionnaire (FFMQ); Resilience Scale for Chinese Adolescents.	Related psychological/ educational outcome	Academic burnout decreased significantly, while dispositional mindfulness and psychological resilience increased significantly in the experimental group compared with the control group.	Mean effect size ($d = 0.03$).

Lowe and Wuthrich (2021) [45] Australia	56 students (43 female, 13 male) in their final year of secondary school (Grade 12). Four schools. Age range: 17–18 years.	Pilot programme. Randomised controlled trial. Control group (n = 28). Eight weekly 1-h sessions. Pretest, posttest, and 3-month follow-up.	"Study Without Stress" (SWOS) programme designed to reduce student stress. CBT skills including psychoeducation, goal setting, time management, cognitive restructuring, problem-solving, management of perfectionism and procrastination, sleep hygiene, relaxation, and examination preparation. Standardised programme with homework exercises. Face-to-face groups of 3–10 students. Delivered by teachers and school counsellors.	Academic-stress target	Dependent variables: psychological distress (stress, depression, and anxiety), self-efficacy, and teacher-reported difficulties. Measures: DASS-21; Self-Efficacy Questionnaire for Children (SEQ-C); Strengths and Difficulties Questionnaire (SDQ), teacher version. Intention-to-treat analysis using hierarchical mixed models.	Related psychological/ educational outcome	Significant reductions in stress and overall distress and increases in total, academic, and emotional self-efficacy from pretreatment to posttreatment compared with the control group. Improvements were maintained over time. No significant reduction in symptoms of depression or anxiety compared with the control group.	Stress ($g = 0.13$), anxiety ($g = 0.23$), self-efficacy ($g = 0.17$), and emotional self-efficacy ($g = 0.33$). No significant effect over time on emotional problems.
Melhe et al. (2021) [46] Jordan	Male Grade 7 students with low academic achievement (n = 28).	Randomised controlled pretest–posttest study. Purposive sample; participants were randomly assigned to experimental and control groups (n = 14 per group)	Training programme based on positive thinking. Face-to-face delivery.	Academic-stress target	Dependent variables: psychological resilience and academic stress. Measures: Psychological Resilience Scale; Academic Stress Scale.	Direct academic/school-related stress outcome	Improvements in psychological resilience and academic stress in the experimental group compared with the control group.	Psychological resilience ($Z = -2.620$), examination stress ($Z = -2.249$), and personal stress ($Z = -2.232$) showed significant improvements. Significant improvements were also reported for the other dimensions of the Academic Stress Scale.

Nadaf et al. (2024) [47] India	Secondary and upper-secondary students with learning difficulties (n = 100).	Mixed-methods research design. Quantitative component: quasi-experimental pretest–posttest design. No control group. Duration: 12 weeks, twice weekly.	Life-skills training programme covering self-awareness, emotional regulation, problem-solving, and effective communication. Face-to-face delivery by teachers and school counsellors.	Academic-stress target	Dependent variable: academic stress. Measures: Academic Stress Scale (ASS); Life Skills Assessment Scale (LSAS); semi-structured focus-group interviews with students, teachers, and families.	Direct academic/school-related stress outcome	Significant reduction in academic stress following the programme. Improvements in coping strategies, self-esteem, emotional regulation, problem-solving skills, communication, self-efficacy, emotional support, resilience, self-awareness, and academic performance.	Statistically significant reduction in stress levels ($t = 10.56$, $p < 0.001$).
Omeke et al. (2024) [48] Nigeria	Secondary school students from rural areas (n = 94), with high academic stress scores and indicators of school maladjustment. Age range: 11–17 years.	Randomised controlled pretest–posttest study. Control group (n = 47)	Rational Emotive Behaviour Therapy (REBT) programme involving cognitive alliance, awareness and time management, cognitive disputation and restructuring, reframing, problem-solving skills, and reinforcement. Face-to-face delivery.	Academic-stress target	Dependent variables: academic stress and school adjustment. Measures: Academic Adjustment Scale; modified version of the Academic Stress Scale for Students.	Direct academic/school-related stress outcome	Significant effect on the management of academic stress. No significant effect on improving academic adjustment.	Academic stress decreased ($d = 1.75$). For school adjustment, the reported effect size was $d = 0.44$ at post-intervention and $d = 0.52$ at follow-up.
Pires et al. (2026) [49] Portugal	Students in Grades 9–12 (n = 35). Age range: 13–18 years. Mean age: 15.63 years. High levels of test anxiety.	Randomised pilot trial. Control group (n = 18). Conducted during the 2021/2022 academic year. Twelve weekly individual 90-min sessions. Pretest, posttest, and 3-month follow-up.	Mindfulness. Acting with Acceptance, Mindfulness and Compassion to Cope with Test and Examination Anxiety (AcAdeMiC) programme. Four sequential modules. Online delivery via Zoom by a therapist, outside school hours.	Academic-demand target	Dependent variables: test anxiety and self-compassion. Measures: Reactions to Tests (RTT); External and Internal Shame Scale (EISS); Mental Health Continuum–Short Form (MHC-SF); Forms of Self-Criticising/Attacking and Self-Reassuring Scale (FSCRS); Avoidance and Fusion Questionnaire for Youth (AFQ-Y); Test Anxiety Acceptance and Action Questionnaire for Adolescents (TA-AAQ-A); Child and Adolescent Mindfulness Measure (CAMM); Self-Compassion Scale (SCS); Social	Related psychological/educational outcome	Test anxiety decreased and self-compassion increased in the experimental group during treatment compared with the control group.	Test anxiety and self-compassion improved compared with the control group ($d = 0.40$). Within the experimental group, a significant change was observed over treatment ($\eta p^2 = 0.57$).

					Anxiety Session Change Index (SASCI); Clinical Global Impression–Social Anxiety (CGI-SA).			
Puolakanaho et al. (2019) [50] Finland	Grade 9 secondary school students (n = 243). Mean age: 15.27 years. Half of the participants had academic difficulties.	Randomised controlled longitudinal trial. Control group (n = 82). Duration: 5 weeks. Pretest and posttest.	Youth COMPASS programme based on Acceptance and Commitment Therapy, including acceptance skills, mindfulness, self-compassion, and adaptation. Digital delivery via the Internet and mobile devices; one group additionally attended face-to-face meetings. Participants had to complete at least six 5–10-min exercises each week.	Academic-demand target	Dependent variables: general stress, school stress, and academic buoyancy. Measures: single item for general stress ("Do you feel this type of stress at this moment?"); adapted scale from the Health Behaviour in School-aged Children (HBSC) study; Academic Resilience Scale. Intervention adherence was assessed through the number of completed tasks.	Direct academic/school-related stress outcome	Statistically significant reduction in general stress and increased academic buoyancy compared with the control group. No clear changes in school stress, although a trend was observed among male students. Participants with higher baseline general and school stress benefited more. Differences in academic skills did not influence intervention outcomes.	General stress (d = 0.22); school stress (d = 0.18); academic buoyancy (d = 0.27).
Sellakumar (2015) [51] India	Students in Grades 6–12 (n = 100). One school. Age range: 11–17 years.	Randomised controlled pretest–posttest study. Participants were randomly assigned to experimental and control groups (n = 50 per group).	Slow deep-breathing exercise aimed at reducing anxiety, with academic and test anxiety conceptualised as forms of situational anxiety. Face-to-face delivery.	Academic-demand target	Dependent variable: anxiety (state, trait, and general). Measure: State–Trait Anxiety Inventory.	Related psychological/educational outcome	Significant reductions in state, trait, and overall anxiety following slow deep-breathing exercises. Significant time × group interactions were found for all three anxiety outcomes. State-anxiety scores decreased more than trait-anxiety scores.	Time × group interaction effect sizes: $\eta p^2 = 0.464$ for state anxiety, $\eta p^2 = 0.362$ for trait anxiety, and $\eta p^2 = 0.567$ for overall anxiety.

Ulaş and Seçer (2022) [52] Türkiye	Grade 8 secondary school students experiencing school burnout (n = 30). Two schools.	Experimental mixed-methods research design. Control group (n = 15). Duration: 9 weeks. Assessment before, during, and after the intervention.	CBT-based psychoeducation. Three themes: factors predisposing students to school burnout (social, school, and classroom factors); symptoms of school burnout (school-related and personal); and protective factors (social support resources, school–family cooperation, enrichment of the learning environment, and positive teacher approaches). Face-to-face delivery.	Academic-demand target	Dependent variable: school burnout (emotional exhaustion, depersonalisation, and reduced personal accomplishment). Measures: School Burnout Scale; two Semi-Structured Interview Protocols addressing school burnout before the programme and programme evaluation; Session Evaluation Protocol.	Related psychological/ educational outcome	Effective in reducing school burnout. Significant reductions in emotional exhaustion, depersonalisation, and reduced personal accomplishment in the experimental group.	Emotional exhaustion ($F(1,29) = 5.327$, $p = 0.029$, $\eta^2 = 0.165$); depersonalisation ($F(1,29) = 15.28$, $p = 0.001$, $\eta^2 = 0.361$); personal accomplishment ($F(1,29) = 7.995$, $p = 0.009$, $\eta^2 = 0.228$).
Vestad and Tharaldsen (2022) [53] Norway	Grade 8 secondary school students (n = 545). Eleven schools. Age range: 13–14 years.	Qualitative exploratory research design. No control group. Duration: 2018/2019 school year; 20 sessions of 60 min each.	Social and Emotional Learning (SEL). ROBUST intervention covering five socio-emotional competencies: relationship skills, emotional regulation, mindfulness, growth mindset, and problem-solving. Each topic was addressed in four sessions. Face-to-face delivery during regular school hours by teachers.	Academic-demand target	Dependent variable: academic stress. Measures: three semi-structured, open-ended focus-group interviews (n = 26); participant validation.	Related psychological/ educational outcome	Students perceived mindfulness, problem-solving, and growth mindset as useful tools for coping with academic stress. Emotional regulation and relationship skills were considered more difficult to use, although they were also perceived as beneficial for coping with school stress.	Mindfulness, problem-solving, and growth mindset were perceived as easier to use than relationship skills and emotional regulation.

Wang et al. (2022) [54] China	Primary and secondary school students in Grades 5–8 from 152 schools. Participants included in analyses: n = 28,398. Mean age: 12.28 years.	Two-point repeated cross-sectional survey with a nested longitudinal subsample. No control group. T1: April–May 2021. Policy implementation: July 2021. T2: December 2021.	Organisational changes. "Double Reduction" policy requiring schools to reduce homework burden and extracurricular tutoring to decrease academic stress and promote students' mental health. Face-to-face implementation within schools.	Organisational burden reduction	Dependent variables: depression and anxiety. Measures: Patient Health Questionnaire (PHQ-9); Generalized Anxiety Disorder Scale (GAD-7); Adolescent Self-Rating Life Events Checklist (ASLEC); five questions assessing study and living conditions related to the "Double Reduction" policy (reduced homework, increased extracurricular participation, increased physical activity, more family time, and reduced academic stress), plus one item assessing sleep duration.	Related psychological/ educational outcome	The prevalence of depression and anxiety decreased slightly. Participants whose academic stress decreased were less likely to experience mental health problems. Reduced homework, increased extracurricular activities, more family time, reduced academic pressure, and sleeping at least 8 h were protective factors for mental health.	PHQ-9 and GAD-7 scores showed small changes ($d = 0.030$ and $d = 0.015$, respectively).
Wang et al. (2024) [55] China	Primary and secondary school students in Grades 5–8 ($n = 36{,}637$ completing all measures). Age range: 10–15 years. Mean age: 11.97 years.	Repeated cross-sectional survey with a nested longitudinal subsample. No control group. Duration: one year, with assessments before and after policy implementation	Organisational changes. "Double Reduction" policy aimed at reducing academic burden and promoting students' mental health. Face-to-face implementation.	Organisational burden reduction	Dependent variables: depression and anxiety. Measures: PHQ-9; GAD-7; ASLEC; five questions related to the "Double Reduction" policy (reduced homework, access to more extracurricular activities, increased physical activity, more family time, and reduced academic stress), plus sleep duration.	Related psychological/ educational outcome	Symptoms of depression and anxiety decreased significantly. Attending a private school, reducing homework burden, participating in more extracurricular activities, spending more time with family, reducing academic stress, and sleeping for 8 h were associated with a lower likelihood of mental health problems.	Depressive symptoms ($\chi^2 = 427.80$, $p < 0.001$) and anxiety symptoms ($\chi^2 = 487.14$, $p < 0.001$).
Wang et al. (2026) [56] China	Grade 8 students from one secondary school ($n = 174$) experiencing academic pressure. Five classes. Age range: 14–15 years. Mean	Cluster-randomised controlled study. Five classes randomly assigned to the intervention or control condition. Control group ($n = 77$)	Stress-mindset course ("Learn to Manage Stress Positively"), designed to promote a stress-is-enhancing mindset by recognising the potential benefits of stress. Face-to-face classroom delivery by a teacher.	Academic-demand target	Dependent variables: stress mindset and perceived stress. Measures: Stress Mindset Measure (SMM); Perceived Stress Scale-10; Adolescent Self-Rating Life Events Checklist.	Related psychological/ educational outcome	No significant overall reduction in perceived stress at the group level. The intervention group showed a significantly greater increase in a stress-is-enhancing mindset, which was associated with decreased perceived stress.	The intervention did not have a notable effect on change in perceived stress ($t = 0.41$, $df = 2.39$, $p = 0.715$).

	age: 14.62 years.							
Yale-Soulière et al. (2024) [57] Canada	Grade 10 and 11 secondary school students (n = 48) with high levels of test anxiety. Five schools. Age range: 14–17 years.	Randomised controlled trial. Control group (n = 24). Six 1-h workshops. Pre- and post-intervention assessments and 6-month follow-up.	"Pastel" programme based on a cognitive-behavioural approach combined with study skills: education about test anxiety, self-monitoring, cognitive restructuring, problem-solving, avoidance and exposure, physiological strategies, and organisational and study strategies. Face-to-face delivery in subgroups of 12 students.	Academic-demand target	Dependent variables: test anxiety (primary outcome); social anxiety, generalised anxiety, depressive symptoms, and academic self-efficacy (secondary outcomes). Measures: Friedben Brief Test Anxiety Scale (B-FTAS); Spence Children's Anxiety Scale (SCAS); Depression Anxiety Stress Scales; Self-Efficacy Questionnaire for Children.	Related psychological/ educational outcome	Test anxiety decreased immediately after the intervention compared with the control group, and the improvement was maintained at the 6-month follow-up. Depressive symptoms and social anxiety decreased after the intervention, with changes maintained at 6 months. No significant between-group changes were found in generalised anxiety or academic self-efficacy.	The reported effect size for test anxiety was d = 0.56 between baseline and post-intervention and d = 0.37 at the 6-month follow-up.

Yue et al. (2024) [58] China	Grade 8 secondary school students (n = 202; n = 184 in the final analysis). Six classes from one secondary school. Mean age: 14.14 years.	Quasi-experimental study. Control group (n = 99; n = 89 in the final analysis). Duration: 8 weeks, 6–7 min daily. Assessments before the intervention (Week 1), during the intervention (Week 5), immediately after the intervention (Week 9), and 1 month after the intervention.	Mindfulness. Brief Meditation Intervention (BMI). Four themes: learning to experience breathing meditation, mindful physical relaxation, awareness of the current emotional state, and review and consolidation of meditation techniques. Face-to-face delivery at school, led by the tutor.	Academic-demand target	Dependent variables: test anxiety, mindfulness, and self-efficacy. Measures: Chinese version of the Mindful Attention Awareness Scale (CMAAS); General Self-Efficacy Scale (GSES); Test Anxiety Scale (TAS).	Related psychological/ educational outcome	Significant reduction in test anxiety and increases in mindfulness and self-efficacy over time. Mindfulness and self-efficacy mediated the relationship between BMI and test anxiety. The intervention group showed a significant decrease in test-anxiety levels compared with the control group.	The mediation effect accounted for 68.35%.
